# Adhesion-Regulating Molecule from *Haemonchus contortus*: Potential Antigen for Diagnosis of Early Infection in Goats

**DOI:** 10.3390/pathogens9010034

**Published:** 2019-12-30

**Authors:** Kalibixiati Aimulajiang, Muhammad Ali-ul-Husnain Naqvi, Wen Chu, Mingmin Lu, Xiaowei Tian, Yongqian Bu, Muhammad Ali Memon, Xiangrui Li, Lixin Xu, Xiaokai Song, Ruofeng Yan

**Affiliations:** MOE Joint International Research Laboratory of Animal Health and Food Safety, College of Veterinary Medicine, Nanjing Agricultural University, Nanjing 210095, China; 2017207022@njau.edu.cn (K.A.); 2017207047@njau.edu.cn (M.A.-u.-H.N.); 2017107076@njau.edu.cn (W.C.); 2015207018@njau.edu.cn (M.L.); 2017207011@njau.edu.cn (X.T.); 2016207023@njau.edu.cn (Y.B.); 2016207040@njau.edu.cn (M.A.M.); lixiangrui@njau.edu.cn (X.L.); xulixin@njau.edu.cn (L.X.); songxiaokai@njau.edu.cn (X.S.)

**Keywords:** *Haemonchus contortus*, adhesion regulating molecule, serological diagnosis, Western blotting, indirect ELISA

## Abstract

*Haemonchus contortus*, a blood-sucking nematode of ruminants, causes large economic losses worldwide. Diagnosis of infection mainly depends on the evaluation of clinical signs and fecal examination. However, this has limitations for the diagnosis of early or light infections, where serological diagnosis seems to be more accurate and reliable. In this study, the recombinant *H. contortus* adhesion-regulating molecule protein (rHCADRM) was expressed and purified, and its diagnostic potential was evaluated. Serum samples from goats experimentally infected with *H. contortus* (*n* = 5) were collected at 0 (before infection, negative control), 7, 14, 21, 35, 49, 63, 85, and 103 days post-infection (DPI). The reactions between rHcADRM and goat serum were tested using Western blot (WB) analysis. The results show that rHcADRM can be recognized in the serum as early as 14 DPI, and the antibody against rHcADRM in infected goat could be maintained for over 89 days. No reaction was found between rHcADRM and antibodies against *Trichinella spiralis*, *Fasciola hepatica*, or *Toxoplasma gondii*. An indirect enzyme-linked immune sorbent assay (ELISA) was developed based on rHcADRM. The optimal coating antigen (279 ng of rHcADRM/well) and serum dilutions (1:50) were determined by checkerboard titration. A total of 64 serum samples, including 32 from *H. contortus* infection goats and 32 from helminth-free goats, were used to determine the positive (0.362) and negative (0.306) cut-off values for the ELISA. The results show this serological diagnosis method is highly sensitive (90.6%) and specific (93.75%). The coefficient of variation within run and between runs was less than 11%. To apply this indirect ELISA during field examination, 51 serum samples were randomly collected from goat farms and tested using this method. The result showed that 19.6% (10/51) of goats were infected with *H. contortus*, which was 100% consistent with the necropsy result, higher than that of fecal examination (15.7%, 8/51). These results indicate that rHcADRM could be a potential antigen for diagnosis of *H. contortus* infection in goats.

## 1. Introduction

*Haemonchus contortus* is one of the most important gastrointestinal nematodes of small ruminants. This parasitic nematode feeds on the blood from capillaries in the abomasum of sheep, goat, cattle, and other ruminants [1,2]. Infection with this parasite often results in anemia, diarrhea, weight loss, or even death in young animals, which causes large economic losses [3,4]. Mortality ranging from 30% to 50% has been reported in lambs and kids in acute cases [5]. Ruminants are infected by ingestion of the third-stage larva (L3). The worms develop into adults at about 21 days post-infection (DPI) and begin to shed eggs through feces [6].

Clinical signs evaluation and fecal examination are the most common methods for the diagnosis of *H. contortus* infection. However, clinical signs usually become apparent only when the infection is heavy, and eggs can be found in the feces after the prepatent period of approximately 3–4 weeks. Limitations to diagnosis often occur with light or early infection. These traditional diagnostic methods are time consuming and lack the capacity to diagnose the infection, especially during early stages [7,8,9]. Novel diagnosis methods for early and light infections are needed.

Excretory and secretory products (ESPs) are produced and released by parasites during infection [10]. *H. contortus* ESPs (HcESPs) contain many proteins that can perform various functions including modulating the host immune response [11,12]. ESPs were reported as diagnostic antigens for pre-patent detection of *H. contortus* infection in sheep [13]. Adhesion regulating molecule (ADRM) was identified as one of the HcESPs that can be isolated from different larval stages of this parasite; however, its diagnostic potential is still unknown. ADRM is a ubiquitin receptor, a known component of the proteasome [14], which is a potential candidate for immunological applications.

In this study, ADRM was purified and expressed, and antibody detection at different levels of *H. contortus* infection was evaluated using Western blotting. Indirect ELISA was established and optimized based on the HcADRM antigen. The diagnostic potential of HcADRM was evaluated using sera samples collected from the field.

## 2. Results

### 2.1. Expression and Purification of rHcADRM

**Figure 1:**
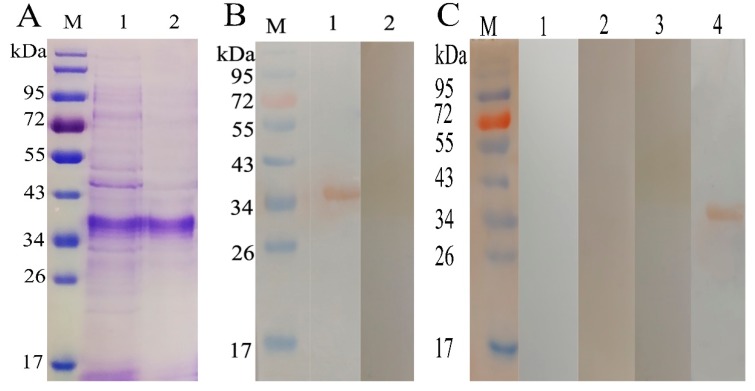
(**A**): SDS-PAGE. Lane M: Standard protein molecular weight marker, lane 1: rHcADRM was expressed after induction with IPTG, Lane 2: Purified rHcADRM. (**B**): Western blot. Lane 1: rHcADRM was recognized by serum from goat infected with *H. contortus*; lane 2: No reactions between rHcADRM and normal goat serum (negative control). (**C**): Western blot to evaluate the specificity of rHcADRM. Lane 1: No reaction with antibody against F. hepatica; lane 2: No reaction with antibody against *T. gondii*; lane 3: No reaction with antibody against *T. spiralis*; lane 4: Positive reaction with antibody against *H. contortus* (positive control).

Sodium dodecyl sulfate-polyacrylamide gel electrophoresis (SDS-PAGE) showed that *H. contortus* adhesion-regulating molecule protein rHcADRM is expressed as a fusion protein, and a band size of 35 kDa was found after purification (Figure 1A). The calculated molecular mass of the HcADRM protein was its original size of 29 kDa after reduction of the His-tagged fusion protein of the pET-28a vector (6 kDa).Western blotting (WB) showed that the rHcADRM could be recognized in the serum from goats infected with *H. contortus* (Figure 1B); however, no cross-reaction was found between rHcADRM and antibody against *Trichinella spiralis*, *Fasciola hepatica*, or *Toxoplasma gondii* (Figure 1C).

### 2.2. Potential of rHcADRM in Diagnosis of H. contortus Infection

**Figure 2:**
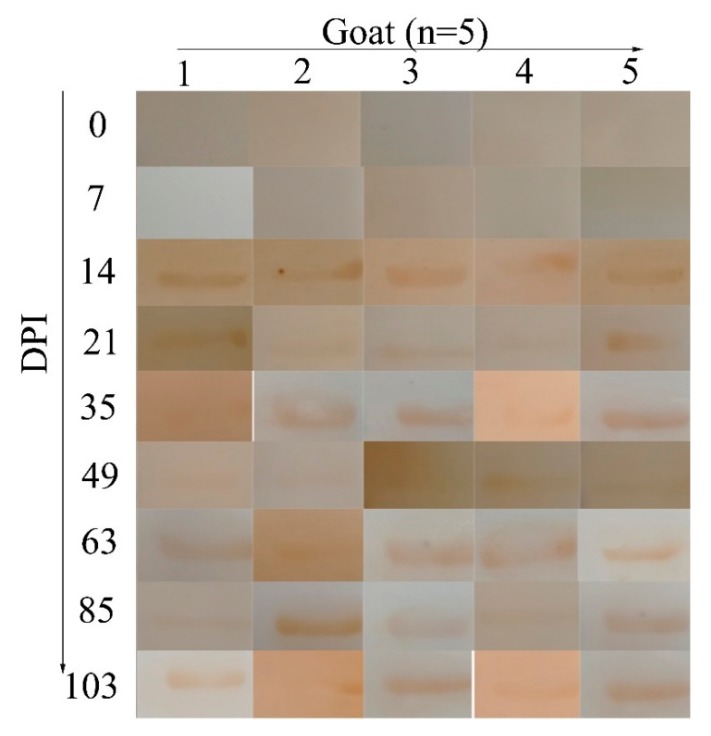
Western blots showing the reactions between rHcADRM and serum from goats (*n* = 5) infected with *H. contortus*. X axis: 1, 2, 3, 4 and 5 represent five goats. Y axis: Serum collected at 0, 7, 14, 21, 35, 49, 63, 85 and 103 days post infection.

The results of WB (Figure 2) showed that rHcADRM could be recognized in the serum from goats infected with *H. contortus* at 14 DPI, which persisted until the end of the experiment (103 DPI), whereas the serum from goats before infection and 7 DPI did not react with rHcADRM. The results are summarized in Table S1, which indicate that rHcADRM has a potential value for the early diagnosis of *H. contortus* infection.

### 2.3. Indirect ELISA Constructed Based on rHcADRM

**Figure 3:**
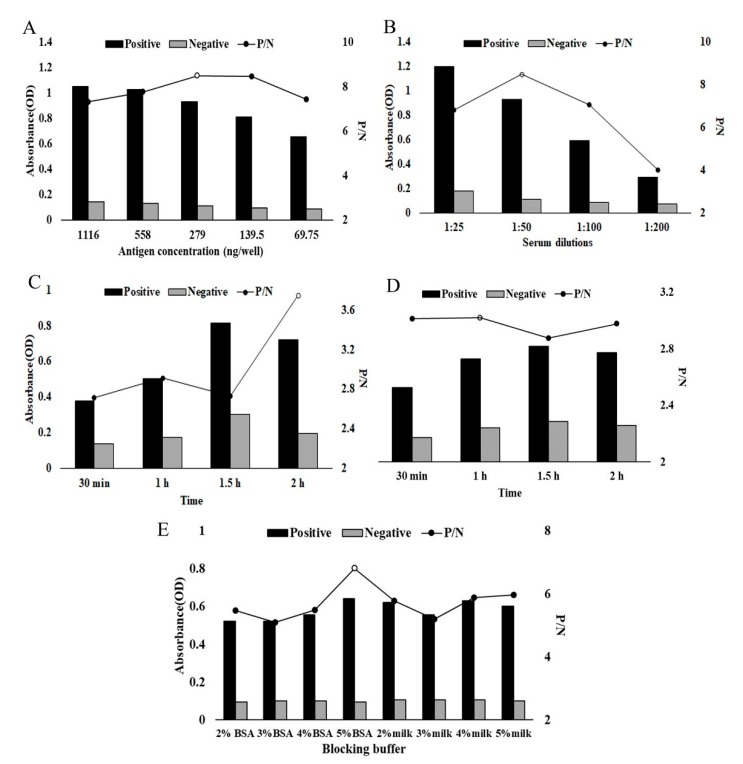
Optimization of indirect ELISA using rHcADRM as coating antigen. (**A**) Coating antigen (69.76, 139.5, 279, 558 and 1116 ng of rHcADRM per well). (**B**) Dilution of first antibody (1:25, 1:50, 1:100, 1:200). (**C**) Incubation time for first antibody (30 min, 1 h, 1.5 h and 2 h). (**D**) Incubation time for second antibody (30 min, 1 h, 1.5 h and 2 h). (**E**) Blocking buffer (2%, 3%, 4%, 5% BSA and 2%, 3%, 4%, 5% skim milk).

The optimal concentration of rHcADRM for coating was determined to be 279 ng/well (Figure 3A), and the serum (first antibody) diluted at 1:50 was selected based on checkerboard titrations (Figure 3B). Other factors, like incubation time for first antibody (2 h, Figure 3C), second antibody (1 h, Figure 3D), and blocking buffer (5% Bovine Serum Albumin, BSA, Figure 3E), were optimized, which gave the highest Positive/Negative values.

### 2.4. Sensitivity, Specificity, and Stability

**Figure 4:**
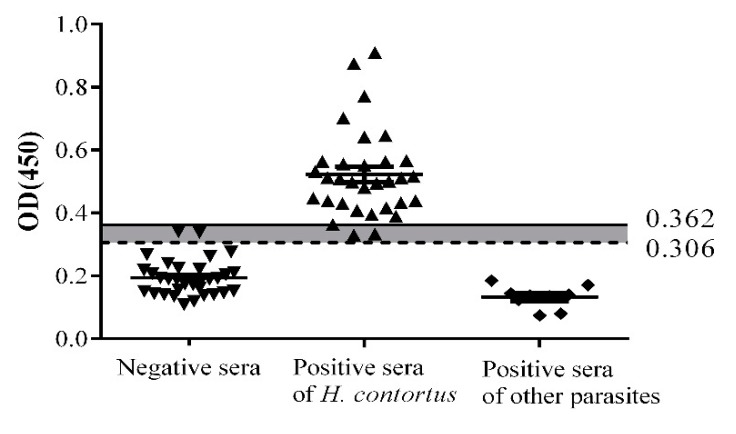
Sensitivity, specificity and cross reactivity of the ELISA. Solid horizontal line represents the positive cut off value (0.362) and dotted horizontal line represents the negative cut off value (0.306). Three samples were observed as false negative and two as false positive.

The sensitivity and specificity of indirect ELISA are shown in Figure 4. Optical Density 450 nm (OD_450_) values of three positive and two negative samples were between the negative and positive cut-offs (0.306 < OD_450_ < 0.362); these samples were considered as false positives and false negatives, respectively. The sensitivity of indirect ELISA identified by positive serum samples was 90.6% (29/32), and the specificity using negative sera was 93.75% (30/32). No cross-reaction was found between rHcADRM and antibodies against *T. spiralis* (*n* = 4), *F. hepatica* (*n* = 1), or *T. gondii* (*n* = 4). The coefficients of variation (CVs) within-run and between runs ranged from 1.49% to 10.35%, which were more accurate than the prior precision criterion of 11% (Table S2).

### 2.5. Diagnosis of H. contortus Infection in the Field

**Figure 5:**
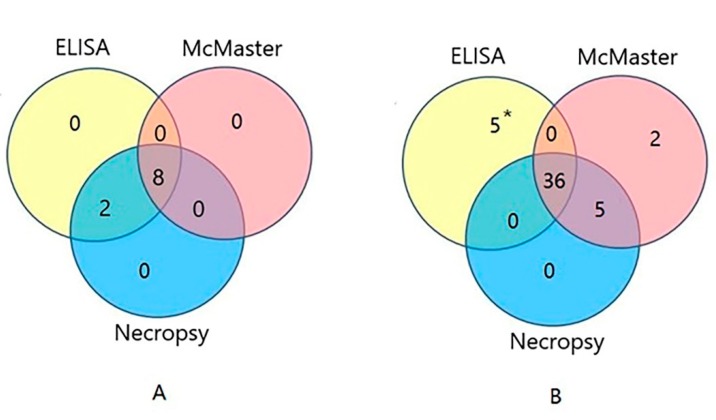
Venn diagram showing the positive (**A**) and negative (**B**) samples tested by ELISA, Mc Master and Necropsy. The 5* represents five samples were found false positive/negative by ELISA.

Infections of 51 goats were tested by indirect ELISA, McMaster, and necropsy (Table S3). The results of indirect ELISA showed that 10 goats were positive, 36 were negative, and 5 were false negative/positive. As shown in Figure 5A, the results of indirect ELISA and necropsy were 100% consistent with each other. However, the McMaster result showed that 15.7% (8/51) goats were infected with *H. contortus*, which was lower than that indicated by indirect ELISA and necropsy testing (19.6%, 10/51). Comparing the results of these three methods, 36 goats were found to be negative for *H. contortus* infection (Figure 5B). The coincidence rate between the indirect ELISA and necropsy was 90.2% ((10 + 36)/51).

## 3. Discussion

*H. contortus* is the dominant nematode parasite in most goat farms [15]. A fast, simple, and accurate method for diagnosis of *H. contortus* infection is important for goat husbandry. Fecal egg examination is a routine procedure, which is not able to indicate the precise level of infection in the early stages. Serological methods have become important for the diagnosis of parasites as they are more accurate in comparison with conventional egg examination and can be used to quickly diagnose infection [16]. Among these methods, ELISA has been most extensively studied as it allows the simultaneous and quick examination of a large number of samples. It is also highly sensitive for the rapid diagnosis of the disease [17]. Many factors affect egg excretion of the parasites and the number of eggs per gram of feces. As such, the eggs per gram (EPG) method is inconvenient and unreliable, indicating the necessity of the use of serological methods.

One important factor to consider for serological diagnosis is cross-reaction [18]. Serum immunoglobin G (IgG) antibodies to other parasite antigens may falsely react with the recombinant antigen of *H. contortus*, and the antibodies to *H. contortus* cross-react with antigens of other parasites. Ideally, a recombinant antigen would be preferred for screening purposes; due to its amenability to mass production, the ADRM protein may be a potential diagnostic antigen for clinical research. Cross-reactivity among adult *H. contortus*, *Moniezia expansa,* and *Fasciola* spp. was reported, and *H. contortus* antigen was found to demonstrate prominent cross-reactivity with other cestodes and trematodes represented by *M. expansa* and *Fasciola* spp., respectively [19]. In contrast, the results of our study revealed that rHcADRM does not demonstrate any cross-reactivity towards commonly found pathogens in goats (*T. spiralis*, *F. hepatica,* and *T. gondii*). This might be due to the differences in the antigens used for our study. As such, recombinant HcADRM might be a potential candidate as a sero-diagnostic reagent.

Antibody detection has been shown to be more sensitive than traditional parasitological microscopic techniques. It is needed in areas characterized by a low level of transmission, low prevalence, and particularly low intensity [20,21]. ESPs are released into the blood circulation and may induce the production of antibodies by infected hosts [22]. These antigens are highly specific and sensitive as diagnostic tools [23,24]. Antibodies can be detected earlier than crude parasite antigens [7,24]. However, the serodiagnostic role of rHcADRM protein has not yet been reported. Thus, we aimed to evaluate the potential serodiagnostic role of rHcADRM during early infection in goats. In this study, WB analysis was used for antibody detection; it showed that the rHcADRM antigen was first detected at 14 DPI and persisted until 103 DPI. However, the anti-ADRM antibodies were not detected in uninfected (0 day) serum. In contrast, a previous study could not detect the infection at the early stage; *H. contortus* eggs were detected in sheep at 21–25 DPI [25]. The evaluated rHcADRM might be a useful antigen for the early diagnosis of *H. contortus*.

As the most sensitive immunoassays, ELISAs offers commercial value in laboratory research and diagnosis of disease biomarkers [26]. We developed an indirect ELISA based on rHcADRM to evaluate its diagnostic potential and to complement the results obtained through Western blotting for further supporting the diagnosis of *H. contortus* infection. WB analysis was used to confirm ELISA-positive sera and invalidate the false positive results, which cause inconveniences for diagnosis [27]. The association of ELISA and WB is a gold standard in human medicine [28] and might also be an effective combination for goats. False positive/negative results are undesirable, and false positive/negative reactions caused by the sample must be eliminated to assay antibodies accurately [29]; so, standardization of the indirect ELISA was performed at first. The results indicated that rHcADRM showed the largest P/N value as well as specificity of the test when the concentration of the coating antigen was adjusted to 279 ng per well and the serum sample was diluted to 1:50. Blocking buffers might eliminate the false positive and negative reactions involved in ELISA, so different blocking buffers and their concentrations were investigated. We found that 5% BSA was the optimal blocking solution. When the incubation time of serum was 120 min and the incubation time of the second antibody was 60 min, the P/N value was the most suitable. Other studies revealed that BSA is one of the most commonly used blocking agents for ELISA, and the incubation time of the buffer significantly affects the performance of the assay [30,31].

In our previous study, we reported an 87% diagnostic sensitivity of rHCA59-based indirect ELISA, which is lower than reported here [12]. In the current study, standardized indirect ELISA based on rHcADRM showed the highest sensitivity of 90.6% (30/32) and specificity of 93.75% (29/32). The OD_450_ value of three positive samples and two negative samples were between the negative and positive cut-off values, and these were considered false positive and false negative, respectively. Previously, ELISA based on recombinant *H. contortus* rHcp26/23 reported the highest prevalence percentage (90.8%) and sensitivity (90%), but the false positive numbers in non-infected sheep were high [19]. Stability assessment of indirect ELISA showed CVs (2.9% to 10.3%) were similar to those reported in a previous study (3.3% to 11.1%) [32] and indicated that the indirect ELISA system showed adequate repeatable precision.

When comparing the results of three different diagnostic methods, a 19.6% (10/51) prevalence of *H. contortus* was recorded by indirect ELISA, which was higher than that by fecal examination (15.7%, 8/51). These results proved that indirect ELISA has better ability to detect *H. contortus* infection compared to McMaster. The presence of worms in goat abomasum confirmed the accuracy of ELISA. However, further studies with large sample numbers are required to improve these assays.

## 4. Materials and Methods 

### 4.1. Expression and Purification of rHcADRM

Recombinant plasmid expression HcADRM (pET-28a (+)/HcADRM; Uniprot: W6NKS2) was provided by Ministry of Education (MOE) joint international Research Laboratory of Animal Health and Food Safety, College of Veterinary Medicine, Nanjing Agriculture University (Nanjing, Jiangsu, China). *Escherichia coli* BL21 (Vazyme Biotech, Nanjing, Jiangsu, China) transformed with pET-28a (+)/HcADRM was induced, and the recombinant protein was purified as previously described [33]. Briefly, the recombinants were incubated at 37 °C until the optical density at 600 nm (OD_600_) of the culture reached 0.6. Isopropyl β-D-1-thiogalactopyranoside (IPTG; Sigma Aldrich, Shanghai, China) was added with the final concentration of 1 mM, followed by incubation for another 5 h. The expression of HcADRM was analyzed by 12% (*w*/*v*) SDS-PAGE. Recombinant protein was purified from the bacterial lysates using Ni^2+^ nitrilotriacetic acid column (GE Healthcare, Pittsburgh, PA, USA) according to the manufacturer’s instructions. The concentration of the purified rHcADRM was determined using a Bradford assay [34].

### 4.2. Parasite, Goat, and Serum Sample

*H. contortus* L3 was maintained by the MOE joint international Research Laboratory of Animal Health and Food Safety, College of Veterinary Medicine, Nanjing Agriculture University (Nanjing, Jiangsu, China).

Five crossbred goats, 4–6 months old, were bought from a goat farm of Xuyi county, Jiangsu province, China. These goats were kept in the animal house of Nanjing Agricultural University. Goats were confirmed to be without helminth infection by feces examination for 4–5 weeks in 2-day intervals. Then, the animals were experimentally infected with 8000 *H. contortus* L3. Serum samples were collected from each goat before infection (negative control) and at 7, 14, 21, 35, 49, 63, 85, and 103 DPI.

We collected 32 positive serum samples from *H. contortus* infected goats and 32 negative serum samples from non-infected goats from another independent experiment.

Serum samples infected with *Fasciola hepatica* were provided by Professor Huang, Yangzhou University (Yangzhou, Jiangsu, China). Serum samples with *Toxoplasma gondii* and *Trichinella spiralis* were provided by the MOE joint international Research Laboratory of Animal Health and Food Safety, College of Veterinary Medicine, Nanjing Agriculture University (Nanjing, Jiangsu, China).

### 4.3. Western Blot Analysis

The purified rHcADRM was separated on SDS-PAGE and transferred to nitrocellulose filter membrane (NC, Merck Millipore, Tullagreen, Carrigtwohill, Ireland). The membrane was blocked with 5% skimmed milk (BD, Baltimore, MD, US) in TBS-T (Tris buffered saline with 0.05% Tween 20) for 1 h at 37 °C and probed with goat serum diluted to 1:100 with TBS-T for 2 h at 37 °C. The membrane was washed 5 times (5 min each) with TBS-T and incubated with rabbit anti-goats IgG antibody conjugated with horseradish peroxidase (HRP) (Thermo Fischer Scientific, Waltham, MA, US) diluted by 1:5000 in blocking buffer for 1 h at 37 °C. The protein band was revealed by using 3,3-diaminobenzidine tetrahydrochloride (DAB, Tiangen Biotech, Beijing, China) substrate as the chromogenic substrate.

### 4.4. Development of Indirect ELISA

Indirect ELISA was optimized to obtain the maximum optical density (OD) with the lowest background [35]. Checkerboard titration was used to optimize the best working dilution of rHcADRM (69.75 to 1116 ng/well) and serum samples (1:25 to 1:200 dilutions). Incubation time (30 min to 2 h) for serum (first antibody), rabbit anti-goat IgG conjugated with HRP (second antibody), and blocking buffers (2% to 5% of skim milk and bovine serum albumin (BSA, Sunshine Bio, Nanjing, Jiangsu, China) was also optimized. Optical density was measured at 450 nm (OD_450_) with a microplate reader (Thermo Fischer Scientific, Waltham, MA, USA). The highest ratio between positive and negative samples was selected as a standard for subsequent runs.

### 4.5. Determination of Cut-Off Value

Indirect ELISA was performed as follows: 279 ng of rHcADRM diluted with 100 μL of coating buffer (0.05 M carbonate buffer, pH 9.6) was incubated at 4 °C overnight in 96-well ELISA plates (Costar, Bodenheim, Germany). After three washes with TBS-T, the plates were blocked with 5% BSA for 1 h at 37 °C, followed by three washes with TBS-T. Then, 100 μL of positive and negative goat sera, diluted in blocking buffer, was added to the wells in triplicate, followed by incubation for 2 h at 37 °C. After three washes, rabbit anti-goat IgG conjugated with HRP (Thermo Fischer Scientific, Waltham, MA, USA) was added and incubated for 1 h at 37 °C. Subsequently, the plates were washed five times, and the peroxidase reaction was visualized using 100 μL/well ready-to-use tetramethylbenzidine-hydrogen peroxide (TMB) solution as a substrate for 10 min at room temperature and stopped by adding 100 μL/well 0.5 M H_2_SO_4_. OD_450_ was measured with a microplate reader (Thermo Fischer Scientific, Waltham, MA, USA).

The negative cut-off value was determined by taking the mean OD_450_ of the negative samples plus 2 multiplied by the standard deviation (SD) [36] and the positive cut-off value by taking the mean OD_450_ of negative samples plus 3 multiplied by SD [37]. For the interpretation, any goat sera that had an OD_450_ greater than positive cut-off point were considered sero-positive, whereas OD_450_ values lower the than the negative cut-off value were considered sero-negative. The intervals between negative and positive cut-off values were considered false results.

### 4.6. Determination of Sensitivity, Specificity, and Stability

We evaluated 32 serum samples from goats infected with *H. contortus* and 32 from non-infected goats to calculate sensitivity and specificity using formulas described previously [38].

A total of nine serum samples infected with *T. spiralis* (*n* = 4), *F. hepatica* (*n* = 1), and *T. gondii* (*n* = 4) were used to evaluate cross-reactivity.

To test the stability of the method, we measured the intra-precision of the indirect ELISA by running three replicates of three positive and three negative sera samples on the same plate. For the inter-precision, three plates were operated and repeated on 3 different occasions. The mean and SD were calculated. The coefficient of variation (CV) of normalized data from the replicates of each control was checked to determine whether it exceeded 10% (expressed as SD/mean × 100%) [35].

### 4.7. Application of the Indirect ELISA

To evaluate the indirect ELISA in field examinations, 51 goats were randomly selected and bought from different goat farms in Nanjing, Jiangsu province, China, to collect the serum samples, and the infection of *H. contorts* was detected by the ELISA. To compare the results with those obtained from fecal examination, fecal samples were collected in parallel and tested using conventional fecal egg counts (FECs) as described previously [39]. Briefly, the McMaster method was performed, 2 g of fecal pellets from each animal was measured, mixed with 58 mL of flotation salt solution, and both chambers of the McMaster slide were filled using a transfer pipette. After setting the slide aside for at least 5 min to allow parasite eggs to float to the surface, all eggs inside the grid areas were counted under a microscope using 10× magnification. The total egg count was (chamber 1 + chamber 2) multiplied by 100 = EPG. The presence of *H. contortus* eggs was determined based on their characteristic shape, dark brown blastomeres, and body dimensions (average length  =  70  ± 10 μm and width  =  45  ±  5 μm) [39,40]. To confirm *H. contortus* infection for these goats, animals were slaughtered humanely, the worms in the abomasum were checked, and the numbers were counted. The treatments of animals in our research were in conformity with the guidelines of the Animal Ethics Committee, Nanjing Agricultural University, China. All animal experiments abided by the guidelines of the Animal Welfare Council of China. The protocols of our experiments were all approved by the Science and Technology Agency of Jiangsu Province. The approval ID is SYXK (SU) 2010-0005.

## 5. Conclusions

We developed an indirect ELISA with the rHcADRM antigen to detect anti-*H. contortus* antibodies in goat serum with good sensitivity and specificity. The Western blot assay developed with rHcADRM was able to detect antibodies during the early (14 DPI) and late stage (21 to 103 DPI) of infection. We conclude that rHcADRM is a potential immunodiagnostic antigen for detecting *H. contortus* infection during the pre-patent and post-patent period in goats. Indirect ELISA based on rHcADRM has the potential to detect *H. contortus* infection from field samples. In the follow-up study, we will use large sample numbers to improve these assays and develop the best product for practical application.

## Supplementary Materials

The following are available online at https://www.mdpi.com/2076-0817/9/1/34/s1, Table S1: Reactions between rHcADRM and serum from goat infected with *H. contortus*, Table S2: Stability of the indirect ELISA, Table S3: Detailed results of field samples tested by indirect ELISA, McMaster and necropsy.
