# Supplementary material for: Adhesion-Regulating Molecule from Haemonchus contortus: Potential Antigen for Diagnosis of Early Infection in Goats
Source: Pathogens. 2019 Dec 30;9(1):34. doi: 10.3390/pathogens9010034 (PMC7168579; doi:10.3390/pathogens9010034)
Supplement: Supplementary file 1 [file pathogens-09-00034-s001.pdf]

Table S1: Reactions between rHcADRM and serum from goat infected with *H. contortus*

DPI	1	2	3	4	5
0	—	—	—	—	—
7	—	—	—	—	—
14	+	+	+	+	+
21	+	+	+	+	+
35	+	+	+	+	+
49	+	+	+	+	+
63	+	+	+	+	+
85	+	+	+	+	+
103	+	+	+	+	+

Reactions between rHcADRM and serum from goat (n=5) infected with *H. contortus* were tested by WB, "+" represent positive reaction and "—" as negative.

Table S2: Stability of the indirect ELISA

**Within-run**	P1	P2	P3	N1	N2	N3
R1	0.662	0.663	0.838	0.209	0.199	0.221
R2	0.580	0.585	0.770	0.205	0.205	0.216
R3	0.578	0.561	0.772	0.216	0.203	0.260
Mean	0.607	0.603	0.793	0.210	0.202	0.232
SD	0.048	0.053	0.039	0.006	0.003	0.024
**CV (%)**	**7.908**	**8.789**	**4.918**	**2.857**	**1.485**	**10.345**

**Between-run**	P4	P5	P6	N4	N5	N6
R-1	0.749	0.683	0.731	0.195	0.133	0.274
R-2	0.725	0.656	0.714	0.199	0.141	0.256

R-3	0.695	0.592	0.643	0.173	0.143	0.275
Mean	0.723	0.644	0.696	0.189	0.139	0.268
SD	0.027	0.047	0.047	0.014	0.005	0.011
**CV (%)**	**3.734**	**7.298**	**6.753**	**7.407**	**3.597**	**4.104**

Positive (P1, P2 and P3) and negative samples (N1, N2 and N3) were tested by indirect ELISA in the same plate by three repeats (R1, R2 and R3). Another positive (P4, P5 and P6) and negative samples (N4, N5 and N6) were tested in three different plates (R-1, R-2 and R-3). The coefficient of variation (CV) was showed as bold.

Table S3: Detailed results of field samples tested by indirect ELISA, McMaster and necropsy

Sample number	ELISA		McMaster		Necropsy	
	OD450	P/N/F	EPG	P/N	Worm counts	P/N
Farm A						
A01	0.139	N	0	N	0	N
A02	0.108	N	0	N	0	N
A03	0.154	N	0	N	0	N
A04	0.341	F	0	N	0	N
A05	0.163	N	0	N	0	N
A06	0.159	N	0	N	0	N
A07	0.278	N	0	N	0	N
A08	0.181	N	0	N	0	N
A09	0.307	F	0	N	0	N
A10	0.302	N	0	N	0	N
A11	0.138	N	0	N	0	N
A12	0.141	N	0	N	0	N
A13	0.422	P	500	P	89	P
A14	0.392	P	400	P	24	P
A15	0.277	N	0	N	0	N

Farm B						
B01	0.18	N	0	N	0	N
B02	0.342	F	0	N	0	N
B03	0.182	N	0	N	0	N
B04	0.152	N	0	N	0	N
B05	0.197	N	0	N	0	N
B06	0.182	N	0	N	0	N
B07	0.144	N	0	N	0	N
B08	0.232	N	0	N	0	N
B09	0.520	P	2200	P	138	P
B10	0.264	N	0	N	0	N
B11	0.156	N	0	N	0	N
B12	0.262	N	0	N	0	N
B13	0.391	P	300	P	33	P
B14	0.462	P	500	P	85	P
B15	0.158	N	0	N	0	N
Farm C						
C01	0.142	N	0	N	0	N
C02	0.526	P	2000	P	182	P
C03	0.19	N	0	N	0	N
C04	0.217	N	0	N	0	N
C05	0.34	F	0	N	0	N
C06	0.195	N	0	N	0	N
C07	0.18	N	0	N	0	N
C08	0.165	N	0	N	0	N
C09	0.327	F	0	N	0	N
C10	0.174	N	0	N	0	N
C11	0.17	N	0	N	0	N
C12	0.369	P	200	P	13	P
C13	0.176	N	0	N	0	N
C14	0.363	P	100	P	4	P
C15	0.164	N	0	N	0	N
C16	0.396	P	0	N	8	P

C17	0.182	N	0	N	0	N
C18	0.203	N	0	N	0	N
C19	0.261	N	0	N	0	N
C20	0.169	N	0	N	0	N
C21	0.380	P	0	N	7	P

P=positive, N=negative, F=false negative / positive
